# Contractive Maps in Locally Transitive Relational Metric Spaces

**DOI:** 10.1155/2014/169358

**Published:** 2014-03-10

**Authors:** Mihai Turinici

**Affiliations:** “A. Myller” Mathematical Seminar, “A. I. Cuza” University, 700506 Iaşi, Romania

## Abstract

Some fixed point results are given for a class of Meir-Keeler contractive maps acting on metric spaces endowed with locally transitive relations. Technical connections with the related statements due to Berzig et al. (2014) are also being discussed.

## 1. Introduction

Let *X* be a nonempty set. Call the subset *Y* of *X*,* almost-singleton* (in short:* asingleton*), provided *y*_1_, *y*_2_ ∈ *Y* implies *y*_1_ = *y*_2_ and* singleton* if, in addition, *Y* is nonempty; note that, in this case, *Y* = {*y*}, for some *y* ∈ *X*. Take a* metric* *d* : *X* × *X* → *R*_+_∶ = [0, *∞*[ over *X*, as well as a self-map *T* ∈ *ℱ*(*X*). (Here, for each couple *A*,  *B* of nonempty sets, *ℱ*(*A*, *B*) denotes the class of all* functions* from *A* to *B*; when *A* = *B*, we write *ℱ*(*A*) in place of *ℱ*(*A*, *A*)). Denote Fix⁡(*T*) = {*x* ∈ *X*; *x* = *Tx*}; each point of this set is referred to as* fixed* under *T*. Concerning the existence and uniqueness of such points, a basic result is the 1922 one due to Banach [1]. Call the self-map *T*, (*d*; *α*)-*contractive* (where *α* ≥ 0), if

(a01) *d*(*Tx*, *Ty*) ≤ *αd*(*x*, *y*), for all *x*, *y* ∈ *X*.

**Theorem 1**

Assume that *T* is (*d*; *α*)-contractive, for some *α* ∈ [0,1[. In addition, let *X* be *d*-complete. Then,

- Fix⁡(*T*) is a singleton, {*z*};
- $T^{n}x\overset{d}{\to }z$ as *n* → *∞*, for each *x* ∈ *X*.

This result (referred to as: Banach's fixed point theorem) found some basic applications to the operator equations theory. As a consequence, a multitude of extensions for it were proposed. Here, we will be interested in the* relational* way of enlarging Theorem 1, based on contractive conditions like

(a02) *F*(*d*(*Tx*, *Ty*), *d*(*x*, *y*), *d*(*x*, *Tx*), *d*(*y*, *Ty*), *d*(*x*, *Ty*), *d*(*y*, *Tx*)) ≤ 0, for all *x*, *y* ∈ *X* with *xℛy*,

where *F* : *R*_+_^6^ → *R* is a function, and *ℛ* is a* relation* over *X*. Note that, when *ℛ* is the* trivial* relation (i.e., *ℛ* = *X* × *X*), a large list of such contractive maps is provided in Rhoades [2]. Further, when *ℛ* is an* order* on *X*, a first result is the 1986 one obtained by Turinici [3], in the realm of ordered metrizable uniform spaces. Two decades after, this fixed point statement was rediscovered (in the ordered metrical setting) by Ran and Reurings [4]; see also Nieto and Rodríguez-López [5]; and, since then, the number of such results increased rapidly. On the other hand, when *ℛ* is an* amorphous* relation over *X*, an appropriate statement of this type is the 2012 one due to Samet and Turinici [6]. The “intermediary” particular case of *ℛ* being* finitely transitive* was recently obtained by Berzig and Karapınar [7], under a class of (*αψ*, *βφ*)-contractive conditions suggested by Popescu [8]. It is our aim in the following to give further extensions of these results, when

- the contractive conditions are taken after the model in Meir and Keeler [9];
- the finite transitivity of *ℛ* is being assured in a “local” way.

Further aspects will be delineated elsewhere.

## 2. Preliminaries 

Throughout this exposition, the ambient axiomatic system is Zermelo-Fraenkel's (abbreviated ZF). In fact, the* reduced* system (ZF-AC + DC) will suffice; here, (AC) stands for the* Axiom of Choice* and (DC) for the* Dependent Choice Principle*. The notations and basic facts to be used in this reduced system are standard. Some important ones are described below.

(A) Let *X* be a nonempty set. By a* relation* over *X*, we mean any nonempty part *ℛ*⊆*X* × *X*. For simplicity, we sometimes write (*x*, *y*) ∈ *ℛ* as *xℛy*. Note that *ℛ* may be regarded as a mapping between *X* and *𝒫*(*X*) (= the class of all subsets in *X*). In fact, denote for *x* ∈ *X*: *X*(*x*, *ℛ*) = {*y* ∈ *X*; *xℛy*} (the* section* of *ℛ* through *x*); then, the desired mapping representation is [*ℛ*(*x*) = *X*(*x*, *ℛ*), *x* ∈ *X*].

Among the classes of relations to be used, the following ones (listed in an “increasing” scale) are important for us:

(P0) *ℛ* is* amorphous*; that is, it has no specific properties at all;
(P1) *ℛ* is an* order*; that is, it is* reflexive* [*xℛx*, ∀*x* ∈ *X*],* transitive* [*xℛy* and *yℛz* imply *xℛz*], and* antisymmetric* [*xℛy* and *yℛx* imply *x* = *y*];
(P2) *ℛ* is a* quasiorder*; that is, it is reflexive and transitive;
(P3) *ℛ* is transitive (see above).

A basic ordered structure is (*N*, ≤); here, *N* = {0,1,…} is the set of natural numbers and (≤) is defined as *m* ≤ *n* if and only if *m* + *p* = *n*, for some *p* ∈ *N*. For each *n* ∈ *N*(1, ≤), let *N*(*n*, >): = {0,…, *n* − 1} stand for the* initial interval* (in *N*) induced by *n*. Any set *P* with *P* ~ *N* (in the sense: there exists a bijection from *P* to *N*) will be referred to as* effectively denumerable*. In addition, given some natural number *n* ≥ 1, any set *Q* with *Q* ~ *N*(*n*, >) will be said to be *n*-*finite*; when *n* is generic here, we say that *Q* is* finite*. Finally, the (nonempty) set *Y* is called (at most)* denumerable* if and only if it is either effectively denumerable or finite.

Given the relations *ℛ*, *𝒮* over *X*, define their* product* *ℛ*∘*𝒮* as

(b01) (*x*, *z*) ∈ *ℛ*∘*𝒮* if there exists *y* ∈ *X* with (*x*, *y*) ∈ *ℛ*, (*y*, *z*) ∈ *𝒮*.

This allows us to introduce the powers of a relation *ℛ* as

(b02) *ℛ* ^0^ = *ℐ*, *ℛ* ^*n*+1^ = *ℛ* ^*n*^∘*ℛ*, *n* ∈ *N*.

(Here, *ℐ* = {(*x*, *x*); *x* ∈ *X*} is the* identical relation* over *X*). The following properties will be useful in the sequel:

(1) $$\begin{array}{c} {ℛ}^{m+n}={ℛ}^{m}\circ {ℛ}^{n},\;{\left({ℛ}^{m}\right)}^{n}={ℛ}^{mn},\;\forall m,n\in N. \end{array}$$

Given *k* ∈ *N*(2, ≤), let us say that *ℛ* is *k*-*transitive*, if *ℛ* ^*k*^⊆*ℛ*; clearly,* transitive* is identical with* 2-transitive*. We may now complete the increasing scale above as

(P4) *ℛ* is* finitely transitive*; that is, *ℛ* is *k*-transitive for some *k* ≥ 2;
(P5) *ℛ* is* locally finitely transitive*; that is, for each (effectively) denumerable subset *Y* of *X*, there exists *k* = *k*(*Y*) ≥ 2, such that the restriction to *Y* of *ℛ* is *k*-transitive;
(P6) *ℛ* is* trivial*; that is, *ℛ* = *X* × *X*; hence, [*xℛy*, ∀*x*, *y* ∈ *X*].

Concerning these concepts, the following property will be useful. Call the sequence (*z*_*n*_; *n* ≥ 0) in *X*, *ℛ*-*ascending*, if *z*_*i*_ *ℛz*_*i*+1_ for all *i* ≥ 0.

**Lemma 2**

Let the *ℛ*-ascending sequence (*z*_*n*_; *n* ≥ 0) in *X* and the natural number *k* ≥ 2 be such that

(b03) *ℛ* is *k*-transitive on *Z* : = {*z*_*n*_; *n* ≥ 0}.

Then, necessarily,

(2) $$\begin{array}{c} \left(\forall r\geq 0\right):\left[\left(z_{i},z_{i+1+r(k-1)}\right)\in ℛ,\forall i\geq 0\right]. \end{array}$$

**Proof**

We will use the induction with respect to *r*. First, by the choice of our sequence, (*z*_*i*_, *z*_*i*+1_) ∈ *ℛ*; whence, the case *r* = 0 holds. Moreover, by definition, (*z*_*i*_, *z*_*i*+*k*_) ∈ *ℛ* ^*k*^; and this, along with the *k*-transitive property, gives (*z*_*i*_, *z*_*i*+*k*_) ∈ *ℛ*; hence, the case of *r* = 1 holds too. Suppose that this property holds for some *r* ≥ 1; we claim that it holds as well for *r* + 1. In fact, let *i* ≥ 0 be arbitrary fixed. Again by the choice of our sequence, (*z*_*i*+1+*r*(*k*−1)_, *z*_*i*+1+(*r*+1)(*k*−1)_) ∈ *ℛ* ^*k*−1^, so that, by the inductive hypothesis (and properties of relational product):

(3) $$\begin{array}{c} \left(z_{i},z_{i+1+(r+1)(k-1)}\right)\in ℛ\circ {ℛ}^{k-1}={ℛ}^{k}; \end{array}$$

and this, along with the *k*-transitive condition, gives (*z*_*i*_, *z*_*i*+1+(*r*+1)(*k*−1)_) ∈ *ℛ*. The proof is thereby complete.

(B) Let (*X*, *d*) be a metric space. We introduce a *d*-convergence and *d*-Cauchy structure on *X* as follows. By a* sequence* in *X*, we mean any mapping *x* : *N* → *X*. For simplicity reasons, it will be useful to denote it as (*x*(*n*); *n* ≥ 0) or (*x*_*n*_; *n* ≥ 0); moreover, when no confusion can arise, we further simplify this notation as (*x*(*n*)) or (*x*_*n*_), respectively. Also, any sequence (*y*_*n*_ : = *x*_*i*(*n*)_; *n* ≥ 0) with *i*(*n*) → *∞* as *n* → *∞* will be referred to as a* subsequence* of (*x*_*n*_; *n* ≥ 0). Given the sequence (*x*_*n*_) in *X* and the point *x* ∈ *X*, we say that (*x*_*n*_),  *d*-*converges* to *x* (written as: $x_{n}\overset{d}{\to }x$) provided *d*(*x*_*n*_, *x*) → 0 as *n* → *∞*; that is,

(4) $$\begin{array}{c} \forall \varepsilon >0,\;\exists i=i\left(\varepsilon \right):\;i\leq n⟹d\left(x_{n},x\right)<\varepsilon . \end{array}$$

The set of all such points *x* will be denoted lim⁡_*n*_(*x*_*n*_); note that it is an asingleton, because *d* is triangular symmetric; if lim⁡_*n*_(*x*_*n*_) is nonempty, then (*x*_*n*_) is called *d*-*convergent*. We stress that the introduced convergence concept $\left(\overset{d}{\to }\right)$ does match the standard requirements in Kasahara [10]. Further, call the sequence (*x*_*n*_),  *d*-*Cauchy* when *d*(*x*_*m*_, *x*_*n*_) → 0 as *m*, *n* → *∞*, *m* < *n*; that is,

(5) $$\begin{array}{c} \forall \varepsilon >0,\;\exists j=j\left(\varepsilon \right):\;j\leq m<n⟹d\left(x_{m},x_{n}\right)<\varepsilon . \end{array}$$

As *d* is triangular symmetric, any *d*-convergent sequence is *d*-Cauchy too; but, the reciprocal is not in general true. Concerning this aspect, note that any *d*-Cauchy sequence (*x*_*n*_; *n* ≥ 0) is *d*-*semi-Cauchy*; that is,

(6) d(xn,xn+1)⟶0  (hence,d(xn,xn+i)⟶0,∀i≥1),as  n⟶∞.

But the reciprocal is not in general true.

The introduced concepts allow us to give a useful property.

**Lemma**

The mapping (*x*, *y*) ↦ *d*(*x*, *y*) is *d*-Lipschitz, in the sense

(7) |d(x,y)−d(u,v)|≤d(x,u)+d(y,v),∀(x,y),(u,v)∈X×X.

As a consequence, this map is *d*-continuous; that is,

(8) $$\begin{array}{c} x_{n}\;\overset{d}{⟶}x,\;y_{n}\overset{d}{⟶}y\;imply\;\;d\left(x_{n},y_{n}\right)⟶d\left(x,y\right).\;\; \end{array}$$

The proof is immediate, by the usual properties of the ambient metric *d*(·, ·); we do not give details.

(C) Let (*X*, *d*) be a metric space; and let *ℛ*⊆*X* × *X* be a (nonempty) relation over *X*; the triple (*X*, *d*, *ℛ*) will be referred to as a* relational metric space*. Further, take some *T* ∈ *ℱ*(*X*). Call the subset *Y* of *X*, *ℛ*-*almost-singleton* (in short: *ℛ*-*asingleton*) provided *y*_1_, *y*_2_ ∈ *Y*, *y*_1_ *ℛy*_2_  ⇒  *y*_1_ = *y*_2_ and *ℛ*-*singleton* when, in addition, *Y* is nonempty. We have to determine circumstances under which Fix⁡(*T*) is nonempty; and, if this holds, to establish whether *T* is* fix*-*ℛ*-*asingleton* (i.e., Fix⁡(*T*) is *ℛ*-asingleton) or, equivalently, *T* is* fix*-*ℛ*-*singleton* (in the sense: Fix⁡(*T*) is *ℛ*-singleton); to do this, we start from the working hypotheses:

(b04) *T* is *ℛ*-semi-progressive: *X*(*T*, *ℛ*): = {*x* ∈ *X*; *x* *ℛ* *Tx*} ≠ *∅*;
(b05) *T* is *ℛ*-increasing: *xℛy* implies *Tx* *ℛ* *Ty*.

The basic directions under which the investigations be conducted are described by the list below, comparable with the one in Turinici [11]:

(2a) We say that *T* is a* Picard operator* (modulo (*d*, *ℛ*)) if, for each *x* ∈ *X*(*T*, *ℛ*), (*T* ^*n*^ *x*; *n* ≥ 0) is *d*-convergent.
(2b) We say that *T* is a* strong Picard operator* (modulo (*d*, *ℛ*)) when, for each *x* ∈ *X*(*T*, *ℛ*), (*T* ^*n*^ *x*; *n* ≥ 0) is *d*-convergent and lim⁡_*n*_(*T* ^*n*^ *x*) ∈ Fix⁡(*T*).
(2c) We say that *T* is a* globally strong Picard operator* (modulo (*d*, *ℛ*)) when it is a strong Picard operator (modulo (*d*, *ℛ*)) and *T* is fix-*ℛ*-asingleton (hence, fix-*ℛ*-singleton).

The sufficient (regularity) conditions for such properties are being founded on* ascending orbital* concepts (in short: (a-o)-concepts). Remember that the sequence (*z*_*n*_; *n* ≥ 0) in *X* is called *ℛ*-*ascending*, if *z*_*i*_ *ℛz*_*i*+1_ for all *i* ≥ 0; further, let us say that (*z*_*n*_; *n* ≥ 0) is *T*-*orbital*, when it is a subsequence of (*T* ^*n*^ *x*; *n* ≥ 0), for some *x* ∈ *X*; the intersection of these notions is just the precise one.

(2d) Call *X*, (a-o, *d*)-*complete*, provided (for each (a-o)-sequence) *d*-Cauchy ⇒  *d*-convergent.
(2e) We say that *T* is (a-o, *d*)-*continuous*, if ((*z*_*n*_)=(a-o)-sequence and $z_{n}\overset{d}{\to }z$) imply $Tz_{n}\overset{d}{\to }Tz$.
(2f) Call *ℛ*, (a-o, *d*)-*almost-self-closed*, if: whenever the (a-o)-sequence (*z*_*n*_; *n* ≥ 0) in *X* and the point *z* ∈ *X* fulfill $z_{n}\overset{d}{\to }z$, there exists a subsequence (*w*_*n*_ : = *z*_*i*(*n*)_; *n* ≥ 0) of (*z*_*n*_; *n* ≥ 0) with *w*_*n*_ *ℛz*, for all *n* ≥ 0.

When the orbital properties are ignored, these conventions give us* ascending* notions (in short: a-notions). On the other hand, when the ascending properties are ignored, the same conventions give us* orbital* notions (in short: o-notions). The list of these is obtainable from the previous one; so, further details are not needed. Finally, when *ℛ* = *X* × *X*, the list of such notions is comparable with the one in Rus ([12], Ch 2, Section  2.2): because, in this case, *X*(*T*, *ℛ*) = *X*.

## 3. Meir-Keeler Contractions

Let (*X*, *d*, *ℛ*) be a relational metric space; and let *T* be a self-map of *X*, supposed to be *ℛ*-semi-progressive and *ℛ*-increasing. The basic directions and sufficient regularity conditions under which the problem of determining the fixed points of  *T*  is to be solved were already listed. As a completion of them, we must formulate the specific metrical contractive conditions upon our data. These, essentially, consist in a “relational” variant of the Meir-Keeler condition [9]. Assume that

(c01) *ℛ* is* nonidentical*: $[\tilde{ℛ}:=ℛ\setminus ℐ$ is nonempty].

Note that, by definition, the introduced relation writes

(c02) $x\tilde{ℛ}y$ if and only if [*xℛy*  and  *x* ≠ *y*];

so, $\tilde{ℛ}$ is* irreflexive* [$x\tilde{ℛ}x$ is false, for each *x* ∈ *X*]. Denote for *x*, *y* ∈ *X*

 (c03) *A*_1_(*x*, *y*) = *d*(*x*, *y*), *B*_1_(*x*, *y*) = diam⁡{*x*, *Tx*, *y*, *Ty*},
 
*A*_2_(*x*, *y*) = (1/2)[*d*(*x*, *Tx*) + *d*(*y*, *Ty*)],
 
*A*_3_(*x*, *y*) = max⁡{*d*(*x*, *Tx*), *d*(*y*, *Ty*)},
 
*A*_4_(*x*, *y*) = (1/2)[*d*(*x*, *Ty*) + *d*(*Tx*, *y*)].

Then, let us introduce the functions

 (c04) *B*_2_ = max⁡{*A*_1_, *A*_2_}, *B*_3_ = max⁡{*A*_1_, *A*_3_}, *B*_4_ = max⁡{*A*_1_, *A*_4_},
 
*C*_1_ = max⁡{*A*_1_, *A*_2_, *A*_4_}, *C*_2_ = max⁡{*A*_1_, *A*_3_, *A*_4_},
 
*𝒢* = {*A*_1_, *B*_2_, *B*_3_, *B*_4_, *C*_1_, *C*_2_}, *𝒢*_1_ = {*A*_1_, *B*_2_, *B*_4_, *C*_1_}, *𝒢*_2_ = {*B*_3_, *C*_2_}.

Note that, for each *G* ∈ *𝒢*, we have

(9) $$\begin{array}{c} A_{1}\left(x,y\right)\leq G\left(x,y\right)\leq B_{1}\left(x,y\right),\;\forall x,y\in X. \end{array}$$

The former of these will be referred to as *G* is* sufficient*; note that, by the properties of *d*, we must have

(10) $$\begin{array}{c} x,y\in X,\;x\tilde{ℛ}y⟹G\left(x,y\right)>0. \end{array}$$

And the latter of these means that *G* is* diameter bounded*.

Given *G* ∈ *𝒢*, we say that *T* is* Meir-Keeler*  (*d*, *ℛ*; *G*)-*contractive*, if

(c05) $x\tilde{ℛ}y$ implies *d*(*Tx*, *Ty*) < *G*(*x*, *y*), expressed as *T* is strictly (*d*, *ℛ*; *G*)-nonexpansive;
(c06) for all *ε* > 0, ∃*δ* > 0: [$x\tilde{ℛ}y$, *ε* < *G*(*x*, *y*) < *ε* + *δ*] ⇒  *d*(*Tx*, *Ty*) ≤ *ε*, expressed as *T* has the Meir-Keeler property (modulo (*d*, *ℛ*; *G*)).

Note that, by the former of these, the Meir-Keeler property may be written as

(c07) for all *ε* > 0, ∃*δ* > 0: [$x\tilde{ℛ}y$, *G*(*x*, *y*) < *ε* + *δ*] ⇒  *d*(*Tx*, *Ty*) ≤ *ε*.

In the following, two basic examples of such contractions will be given.

(A) Let *ℱ*(*re*)(*R*_+_) stand for the class of all *φ* ∈ *ℱ*(*R*_+_) with the (strong)* regressive* property: [*φ*(0) = 0; *φ*(*t*) < *t*, for all *t* > 0]. We say that *φ* ∈ *ℱ*(*re*)(*R*_+_) is* Meir-Keeler admissible*, if

(c08) for all *γ* > 0, ∃*β*∈]0, *γ*[, (∀*t*): *γ* ≤ *t* < *γ* + *β*⇒*φ*(*t*) ≤ *γ*; or, equivalently: for all *γ* > 0, ∃*β*∈]0, *γ*[, (∀*t*): 0 ≤ *t* < *γ* + *β*⇒*φ*(*t*) ≤ *γ*.

Now, given *G* ∈ *𝒢*, *φ* ∈ *ℱ*(*R*_+_), call *T*,  (*d*, *ℛ*; *G*, *φ*)*-contractive*, if

(c09) *d*(*Tx*, *Ty*) ≤ *φ*(*G*(*x*, *y*)), for all *x*, *y* ∈ *X*, $x\tilde{ℛ}y$.

**Lemma 4**

Assume that *T* is (*d*, *ℛ*; *G*, *φ*)-contractive, where *φ* ∈ *ℱ*(*re*)(*R*_+_) is Meir-Keeler admissible. Then, *T* is Meir-Keeler (*d*, *ℛ*; *G*)-contractive.

**Proof**

(i) Let *x*, *y* ∈ *X* be such that $x\tilde{ℛ}y$. The contractive condition, and regressiveness of *φ*, yield *d*(*Tx*, *Ty*) < *G*(*x*, *y*), so that, *T* is strictly (*d*, *ℛ*; *G*)-nonexpansive.

(ii) Let *ε* > 0 be arbitrary fixed; and *δ*∈]0, *ε*[ be the number assured by the Meir-Keeler admissible property of *φ*. Further, let *x*, *y* ∈ *X* be such that $x\tilde{ℛ}y$ and *ε* < *G*(*x*, *y*) < *ε* + *δ*. By the contractive condition and admissible property,

(11) $$\begin{array}{c} d\left(Tx,Ty\right)\leq \varphi \left(G\left(x,y\right)\right)\leq \varepsilon , \end{array}$$

so that *T* has the Meir-Keeler property (modulo (*d*, *ℛ*; *G*)).

Some important classes of such functions are given below.

- For any *φ* ∈ *ℱ*(*re*)(*R*_+_) and any *s* ∈ *R*_+_^0^ : = ]0, *∞*[, put
  (c10) Λ_+_ *φ*(*s*) = inf⁡_*ε*>0_Φ(*s*+)(*ε*), where Φ(*s*+)(*ε*) = sup⁡*φ*(]*s*, *s* + *ε*[);
  (c11) Λ^+^ *φ*(*s*) = max⁡{*φ*(*s*), Λ_+_ *φ*(*s*)}.

By this very definition, we have the representation (for all *s* ∈ *R*_+_^0^)

(12)       Λ+φ(s)=inf⁡ε>0⁡ Φ[s+](ε),where  Φ[s+](ε)=sup⁡⁡φ([s,s+ε[).

From the regressive property of *φ*, these limit quantities are finite; precisely,

(13) $$\begin{array}{c} 0\leq \varphi \left(s\right)\leq \Lambda^{+}\varphi \left(s\right)\leq s,\;\forall s\in R_{+}^{0}. \end{array}$$

Call *φ* ∈ *ℱ*(*re*)(*R*_+_),* Boyd-Wong admissible,* if

(c12) Λ^+^ *φ*(*s*) < *s* (or, equivalently: Λ_+_ *φ*(*s*) < *s*), for all *s* > 0.

(This convention is related to the developments in Boyd and Wong [13]; we do not give details). In particular, *φ* ∈ *ℱ*(*re*)(*R*_+_) is Boyd-Wong admissible provided it is upper semicontinuous at the right on *R*_+_^0^:

(14) Λ+φ(s)=φ(s),(or,equivalently:  Λ+φ(s)≤φ(s)),∀s∈R+0.

Note that this is fulfilled when *φ* is continuous at the right on *R*_+_^0^; for, in such a case, Λ_+_ *φ*(*s*) = *φ*(*s*), for all *s* ∈ *R*_+_^0^.

(II) Call *φ* ∈ *ℱ*(*re*)(*R*_+_),* Matkowski admissible* [14], provided
  (c13) *φ* is increasing and *φ* ^*n*^(*t*) → 0 as *n* → *∞*, for all *t* > 0.

(Here, *φ* ^*n*^ stands for the *n*th iterate of *φ*). Note that the obtained class of functions is distinct from the above introduced one, as simple examples show.

Now, let us say that *φ* ∈ *ℱ*(*re*)(*R*_+_) is* Boyd-Wong-Matkowski admissible* (abbreviated: BWM-admissible) if it is either Boyd-Wong admissible or Matkowski admissible. The following auxiliary fact will be useful.

**Lemma 5**

Let *φ* ∈ *ℱ*(*re*)(*R*_+_) be a BWM-admissible function. Then, *φ* is Meir-Keeler admissible (see above).

**Proof**

The former of these is an immediate consequence of definition. And the second one is to be found in Jachymski [15].

(B) Let us say that (*ψ*, *φ*) is a pair of weak generalized altering functions in *ℱ*(*R*_+_), if

(c14) *ψ* is increasing, and [*φ*(0) = 0; *φ*(*ε*) > *ψ*(*ε*) − *ψ*(*ε* − 0), for all *ε* > 0]
(c15) (for  all  *ε* > 0): limsup⁡_*n*_ *φ*(*t*_*n*_) > *ψ*(*ε* + 0) − *ψ*(*ε*), whenever *t*_*n*_ → *ε* + +.

Here, given the sequence (*r*_*n*_; *n* ≥ 0) in *R* and the point *r* ∈ *R*, we denoted

 
*r*_*n*_ → *r*+ (resp., *r*_*n*_ → *r* + +), if *r*_*n*_ → *r* and
 
*r*_*n*_ ≥ *r* (resp., *r*_*n*_ > *r*), for all *n* ≥ 0 large enough.

Given *G* ∈ *𝒢* and the couple (*ψ*, *φ*) of functions in *ℱ*(*R*_+_), let us say that *T* is (*d*, *ℛ*; *G*, (*ψ*, *φ*))-*contractive*, provided

(c16) *ψ*(*d*(*Tx*, *Ty*)) ≤ *ψ*(*G*(*x*, *y*)) − *φ*(*G*(*x*, *y*)), for all *x*, *y* ∈ *X*, $x\tilde{ℛ}y$.

**Lemma 6**

Suppose that *T* is (*d*, *ℛ*; *G*, (*ψ*, *φ*))-contractive, for a pair (*ψ*, *φ*) of weak generalized altering functions in *ℱ*(*R*_+_). Then, *T* is Meir-Keeler (*d*, *ℛ*; *G*)-contractive (see above).

**Proof**

(i) Let *x*, *y* ∈ *X* be such that $x\tilde{ℛ}y$. Then (as *G* is sufficient), *G*(*x*, *y*) > 0, so that (by the choice of our pair), *φ*(*G*(*x*, *y*)) > 0; wherefrom *ψ*(*d*(*Tx*, *Ty*)) < *ψ*(*G*(*x*, *y*)). This via (*ψ* = increasing) yields *d*(*Tx*, *Ty*) < *G*(*x*, *y*), so that *T* is strictly (*d*, *ℛ*; *G*)-nonexpansive.

(ii) Assume by contradiction that *T* does not have the Meir-Keeler property (modulo (*d*, *ℛ*; *G*)); that is, for some *ε* > 0,

(15) ∀δ>0, ∃(xδ,yδ)∈ℛ~:[ε<G(xδ,yδ)<ε+δ,d(Txδ,Tyδ)>ε].

Taking a zero converging sequence (*δ*_*n*_) in *R*_+_^0^, we get a couple of sequences (*x*_*n*_; *n* ≥ 0) and (*y*_*n*_; *n* ≥ 0) in *X*, so as

(16) (∀n):xnℛ~yn, ε<G(xn,yn)<ε+δn, d(Txn,Tyn)>ε.

By the contractive condition (and *ψ* = increasing), we get

(17) $$\begin{array}{c} \psi \left(\varepsilon \right)\leq \psi \left(G\left(x_{n},y_{n}\right)\right)-\varphi \left(G\left(x_{n},y_{n}\right)\right),\;\forall n; \end{array}$$

or, equivalently,

(18) $$\begin{array}{c} \left(0<\right)\;\varphi \left(G\left(x_{n},y_{n}\right)\right)\leq \psi \left(G\left(x_{n},y_{n}\right)\right)-\psi \left(\varepsilon \right),\;\forall n. \end{array}$$

By (16), *G*(*x*_*n*_, *y*_*n*_) → *ε* + +, so that passing to limsup⁡ as *n* → *∞*,

(19) $$\begin{array}{c} \underset{n}{\lim\;\sup}\;\varphi \left(G\left(x_{n},y_{n}\right)\right)\leq \psi \left(\varepsilon +0\right)-\psi \left(\varepsilon \right). \end{array}$$

But, from the hypothesis about (*ψ*, *φ*), these relations are contradictory. This ends the argument.

## 4. Main Result 

Let (*X*, *d*, *ℛ*) be a relational metric space. Further, let *T* be a self-map of *X*, supposed to be *ℛ*-semi-progressive and *ℛ*-increasing. The basic directions and regularity conditions under which the problem of determining the fixed points of *T* is to be solved, were already listed; and the contractive type framework was settled. It remains now to precise the regularity conditions upon *ℛ*. Denote, for each *x* ∈ *X*(*T*, *ℛ*),

(20) $$\begin{array}{c} \text{spec}\left(x\right)=\left\{i\in N\left(1,\leq \right);xℛT^{i}x\right\}\;\left(\text{the}\;\text{spectrum}\;\text{of}\;x\right). \end{array}$$

Clearly, 1 ∈ spec(*x*), but the possibility of spec(*x*) = {1} cannot be removed. This fact remains valid even if *x* ∈ *X*(*T*, *ℛ*) is* orbital admissible*, in the sense [*i* ≠ *j* implies *T* ^*i*^ *x* ≠ *T* ^*j*^ *x*], when the associated orbit *T* ^*N*^ *x* : = {*T* ^*n*^ *x*; *n* ≥ 0} is effectively denumerable. But for the developments below, it is necessary that these spectral subsets of *N* should have a finite Hausdorff-Pompeiu distance to *N*; hence, in particular, these must be infinite. Precisely, given *k* ≥ 1, let us say that *ℛ* is *k*-*semirecurrent* at the orbital admissible *x* ∈ *X*(*T*, *ℛ*), if

 for each *n* ∈ *N*(1, ≤), there exists *q* ∈ spec(*x*) such that *q* ≤ *n* < *q* + *k*.

A global version of this convention is the following: call *ℛ*,* finitely semirecurrent* if, for each orbital admissible *x* ∈ *X*(*T*, *ℛ*), there exists *k*(*x*) ∈ *N*(1, ≤), such that *ℛ* is *k*(*x*)-semirecurrent at *x*.

Assume in the following that

(d01) *ℛ* is finitely semirecurrent and nonidentical.

Our main result in this exposition is the following.

**Theorem 7**

Assume that *T* is Meir-Keeler (*d*, *ℛ*; *G*)-contractive, for some *G* ∈ *𝒢*. In addition, let *X* be (a-o,  *d*)-complete; and one of the following conditions holds:

- *T* is (a-o,  *d*)-continuous;
- *ℛ* is (a-o,  *d*)-almost-self-closed and *G* ∈ *𝒢*_1_;
- *ℛ* is (a-o,  *d*)-almost-self-closed and *T* is (*d*, *ℛ*; *G*, *φ*)-contractive, for a certain Meir-Keeler admissible function *φ* ∈ *ℱ*(*re*)(*R*_+_);
- *ℛ* is (a-o,  *d*)-almost-self-closed and *T* is (*d*, *ℛ*; *G*, (*ψ*, *φ*))-contractive, for a certain pair (*ψ*, *φ*) of weak generalized altering functions in *ℱ*(*R*_+_).

Then *T* is a globally strong Picard operator (modulo (*d*, *ℛ*)).

**Proof**

First, we check the fix-*ℛ*-asingleton property. Let *z*_1_, *z*_2_ ∈ Fix⁡(*T*) be such that *z*_1_ *ℛz*_2_; and assume by contradiction that *z*_1_ ≠ *z*_2_; whence $z_{1}\tilde{ℛ}z_{2}$. From the very definitions above,

(21) A1(z1,z2)=A4(z2,z2)=d(z1,z2),A2(z1,z2)=A3(z2,z2)=0;

whence *G*(*z*_1_, *z*_2_) = *d*(*z*_1_, *z*_2_). This, via *T* being strictly (*d*, *ℛ*; *G*)-nonexpansive, yields an evaluation like

(22) $$\begin{array}{c} d\left(z_{1},z_{2}\right)=d\left(Tz_{1},Tz_{2}\right)<G\left(z_{1},z_{2}\right); \end{array}$$

which is contradictory; hence the claim follows. It remains now to establish the strong Picard property (modulo (*d*, *ℛ*)). The argument will be divided into several steps.

*Part 1.* We firstly assert that

(23) $$\begin{array}{c} G\left(x,Tx\right)=d\left(x,Tx\right),\;\text{whenever}\;x\tilde{ℛ}Tx. \end{array}$$

Let *x* ∈ *X* be such that $x\tilde{ℛ}Tx$. As *T* is strictly (*d*, *ℛ*; *G*)-nonexpansive, one has *d*(*Tx*, *T* ^2^ *x*) < *G*(*x*, *Tx*). On the other hand,

(24) A4(x,Tx) =(12)d(x,T2x) ≤(12)[d(x,Tx)+d(Tx,T2x)] =A2(x,Tx)≤max⁡⁡{d(x,Tx),d(Tx,T2x)} =A3(x,Tx).

This, along with

(25) d(Tx,T2x)<A3(x,Tx) ⟹d(Tx,T2x)<d(x,Tx) ⟹A3(x,Tx)=d(x,Tx),

gives the desired fact.

*Part 2.* Take some *x*_0_ ∈ *X*; and put (*x*_*n*_ = *T* ^*n*^ *x*_0_; *n* ≥ 0). If *x*_*n*_ = *x*_*n*+1_ for some *n* ≥ 0, we are done, so, without loss, one may assume that, for each *n* ≥ 0,

(d02) *x*_*n*_ ≠ *x*_*n*+1_; hence, $x_{n}\tilde{ℛ}x_{n+1}$, *ρ*_*n*_ : = *d*(*x*_*n*_, *x*_*n*+1_) > 0.

From the preceding part, we derive

(26) $$\begin{array}{c} \rho_{n+1}=d\left(Tx_{n},Tx_{n+1}\right)<G\left(x_{n},x_{n+1}\right)=\rho_{n},\;\forall n, \end{array}$$

so that the sequence (*ρ*_*n*_; *n* ≥ 0) is strictly descending. As a consequence, *ρ* : = lim⁡_*n*_ *ρ*_*n*_ exists as an element of *R*_+_. Assume by contradiction that *ρ* > 0; and let *δ* > 0 be the number given by the Meir-Keeler (*d*, *ℛ*; *G*)-contractive condition upon *T*. By definition, there exists a rank *n*(*δ*) such that *n* ≥ *n*(*δ*) implies *ρ* < *ρ*_*n*_ < *ρ* + *δ*; hence (by a previous representation) *ρ* < *G*(*x*_*n*_, *x*_*n*+1_) = *ρ*_*n*_ < *ρ* + *δ*. This, by the Meir-Keeler contractive condition we just quoted, yields (for the same *n*), *ρ*_*n*+1_ = *d*(*Tx*_*n*_, *Tx*_*n*+1_) ≤ *ρ*; contradiction. Hence, *ρ* = 0, so that

(27) $$\begin{array}{c} \rho_{n}∶=d\left(x_{n},x_{n+1}\right)=d\left(x_{n},Tx_{n}\right)⟶0,\;\text{as}\;n⟶\infty ; \end{array}$$

that is, (see above): (*x*_*n*_; *n* ≥ 0) is *d*-semi-Cauchy.

*Part 3.* Suppose that

(d03) there exist *i*, *j* ∈ *N* such that *i* < *j*, *x*_*i*_ = *x*_*j*_.

Denoting *p* = *j* − *i*, we thus have *p* > 0 and *x*_*i*_ = *x*_*i*+*p*_, so that

(28) $$\begin{array}{c} x_{i}=x_{i+np},\;x_{i+1}=x_{i+np+1},\;\forall n\geq 0.\;\; \end{array}$$

By the introduced notations, *ρ*_*i*_ = *ρ*_*i*+*np*_, for all *n* ≥ 0. This, along with *ρ*_*i*+*np*_ → 0 as *n* → *∞*, yields *ρ*_*i*_ = 0, in contradiction with the initial choice of (*ρ*_*n*_; *n* ≥ 0). Hence, our working hypothesis cannot hold; wherefrom

(29) $$\begin{array}{c} \forall i,j\in N:\;i\neq j\;\text{implies}\;x_{i}\neq \;x_{j}.\;\; \end{array}$$

*Part 4*. As a consequence of this, the map *i* ↦ *x*_*i*_ : = *T* ^*i*^ *x*_0_ is injective; hence, *x*_0_ is orbital admissible. Let *k* : = *k*(*x*_0_) ≥ 1 be the semirecurrence constant of *ℛ* at *x*_0_ (assured by the choice of this relation). Further, let *ε* > 0 be arbitrary fixed; and *δ* > 0 be the number associated by the Meir-Keeler (*d*, *ℛ*; *G*)-contractive property; without loss, one may assume that *δ* < *ε*. By the *d*-semi-Cauchy property and triangular inequality, there exists a rank *n*(*δ*) ≥ 0, such that

(30) (∀n≥n(δ)):d(xn,xn+1)<δ4k,whence  d(xn,xn+h)<hδ4k≤δ2,∀h∈{1,…,2k}.

We claim that the following relation holds:

(31) $$\begin{array}{c} \left(\forall s\geq 1\right):\left[d\left(x_{n},x_{n+s}\right)<\varepsilon +\frac{\delta }{2},\forall n\geq n\left(\delta \right)\right]; \end{array}$$

wherefrom, (*x*_*n*_; *n* ≥ 0) is *d*-Cauchy. To do this, an induction argument upon *s* ≥ 1 will be used. The case *s* ∈ {1,…, 2*k*} is evident, by the preceding evaluation. Assume that it holds for all *s* ∈ {1,…, *p*}, where *p* ≥ 2*k*; we must establish its validity for *s* = *p* + 1. As *ℛ* is *k*-semirecurrent at *x*_0_, there exists *q* ∈ spec(*x*_0_) such that *q* ≤ *p* < *q* + *k*; note that the former of these yields (from the *ℛ*-increasing property of *T*), $x_{n}\tilde{ℛ}x_{n+q}$. Now, by the inductive hypothesis and (30),

(32) d(xn,xn+q),d(xn+1,xn+q), d(xn+1,xn+q+1)<ε+δ2<ε+δ,d(xn,xn+1),d(xn+q,xn+q+1)<δ4k<δ<ε+δ.

This, along with the triangular inequality, gives us

(33) d(xn,xn+q+1) ≤d(xn,xn+q)+d(xn+q,xn+q+1) <ε+δ2+δ4k<ε+δ;

wherefrom *B*_1_(*x*_*n*_, *x*_*n*+*q*_) < *ε* + *δ*, so that (by the diameter boundedness property), (0<)  *G*(*x*_*n*_, *x*_*n*+*q*_) < *ε* + *δ*. Taking the Meir-Keeler (*d*, *ℛ*; *G*)-contractive assumption imposed upon *T* into account gives

(34) $$\begin{array}{c} d\left(x_{n+1},x_{n+q+1}\right)=d\left(Tx_{n},Tx_{n+q}\right)\leq \varepsilon , \end{array}$$

so that by the triangular inequality (and (30) again),

(35) d(xn,xn+p+1) ≤d(xn,xn+1)+d(xn+1,xn+q+1)+d(xn+q+1,xn+p+1) <ε+δ4k+kδ4k ≤ε+δ4+δ4=ε+δ2;

and our claim follows.

*Part 5*. As *X* is (a-o,  *d*)-complete, $x_{n}\overset{d}{\to }z$, for some (uniquely determined) *z* ∈ *X*. If there exists a sequence of ranks (*i*(*n*); *n* ≥ 0) with [*i*(*n*) → *∞* as *n* → *∞*] such that *x*_*i*(*n*)_ = *z* (hence, *x*_*i*(*n*)+1_ = *Tz*) for all *n*, then, as (*x*_*i*(*n*)+1_; *n* ≥ 0) is a subsequence of (*x*_*n*_; *n* ≥ 0), one gets *z* = *Tz*. So, in the following, we may assume that the opposite alternative is true:

(d04) ∃*h* ≥ 0: *n* ≥ *h*  ⇒  *x*_*n*_ ≠ *z*.

There are several cases to discuss.

*Case  5a*. Suppose that *T* is (a-o,  *d*)-continuous. Then $y_{n}:=Tx_{n}\overset{d}{\to }Tz$ as *n* → *∞*. On the other hand, (*y*_*n*_ = *x*_*n*+1_; *n* ≥ 0) is a subsequence of (*x*_*n*_); whence $y_{n}\overset{d}{\to }z$; and this yields (as *d* is sufficient), *z* = *Tz*.

*Case  5b*. Suppose that *ℛ* is (a-o,  *d*)-almost-self-closed. Put, for simplicity reasons, *b* : = *d*(*z*, *Tz*). By definition, there exists a subsequence (*u*_*n*_ : = *x*_*i*(*n*)_; *n* ≥ 0) of (*x*_*n*_; *n* ≥ 0), such that *u*_*n*_ *ℛz*, for all *n*. Note that, as lim⁡_*n*_ *i*(*n*) = *∞*, one may arrange for *i*(*n*) ≥ *n*, for all *n*, so that, from (d04),

(36) $$\begin{array}{c} \forall n\geq h:\left[i\left(n\right)\geq h;\;\text{whence}\;\left(\text{see}\;\text{above}\right),\;u_{n}\tilde{ℛ}z\right]. \end{array}$$

This, along with (*Tu*_*n*_ = *x*_*i*(*n*)+1_; *n* ≥ 0) being as well a subsequence of (*x*_*n*_; *n* ≥ 0), gives (via (27) and Lemma 3)

(37) $$\begin{array}{c} A_{1}\left(u_{n},z\right)=d\left(u_{n},z\right)⟶0,\;\;d\left(Tu_{n},z\right)⟶0,\\ d\left(u_{n},Tu_{n}\right)⟶0,\;\;d\left(u_{n},Tz\right)⟶b,\\ d\left(Tu_{n},Tz\right)⟶b; \end{array}$$

whence (by definition)

(38) $$\begin{array}{c} A_{2}\left(u_{n},z\right),A_{4}\left(u_{n},z\right)⟶\frac{b}{2},\\ A_{3}\left(u_{n},z\right),B_{1}\left(u_{n},z\right)⟶b. \end{array}$$

We now show that the assumption *z* ≠ *Tz* (i.e., *b* > 0) yields a contradiction. Two alternatives must be treated.

*Alter 1*. Suppose that *G* ∈ *𝒢*_1_. By the Meir-Keeler contractive condition,

(39) $$\begin{array}{c} d\left(Tu_{n},Tz\right)<G\left(u_{n},z\right)\leq B_{1}\left(u_{n},z\right),\;\forall n\geq h; \end{array}$$

so that, combining with the preceding relations, *G*(*u*_*n*_, *z*) → *b*. This, along with (37) + (38), is impossible for any *G* ∈ *𝒢*_1_; whence, *z* = *Tz*.

*Alter 2*. Suppose that *G* ∈ *𝒢*_2_. The above convergence properties of (*u*_*n*_; *n* ≥ 0) tell us that, for a certain rank *n*(*b*) ≥ *h*, we must have

(40) $$\begin{array}{c} d\left(u_{n},Tu_{n}\right),d\left(u_{n},z\right),d\left(Tu_{n},z\right)<\frac{b}{2},\;\forall n\geq n\left(b\right). \end{array}$$

This, by the *d*-Lipschitz property of *d*(·, ·), gives

(41) $$\begin{array}{c} \left|d\left(u_{n},Tz\right)-b\right|\leq d\left(u_{n},z\right)<\frac{b}{2},\;\forall n\geq n\left(b\right), \end{array}$$

wherefrom, *b*/2 < *d*(*u*_*n*_, *Tz*) < 3*b*/2,  ∀*n* ≥ *n*(*b*). Combining these yields

(42) $$\begin{array}{c} G\left(u_{n},z\right)=b,\;\forall n\geq n\left(b\right),\;\;\forall G\in {𝒢}_{2}. \end{array}$$

Two subcases are now under discussion.

*Alter 2a*. Suppose that *T* is (*d*, *ℛ*; *G*, *φ*)-contractive, for a certain Meir-Keeler admissible function *φ* ∈ *ℱ*(*re*)(*R*_+_). (The case *G* ∈ *𝒢*_1_ was already clarified in a preceding step.) By (42) and this contractive property,

(43) $$\begin{array}{c} d\left(Tu_{n},Tz\right)\leq \varphi \left(b\right),\;\forall n\geq n\left(b\right). \end{array}$$

Passing to limit gives (by (37) above), *b* ≤ *φ*(*b*); contradiction; hence, *z* = *Tz*.

*Alter 2b*. Suppose that *T* is (*d*, *ℛ*; *G*, (*ψ*, *φ*))-contractive, for a certain pair (*ψ*, *φ*) of weak generalized altering functions in *ℱ*(*R*_+_). (As before, the case *G* ∈ *𝒢*_1_ is clear, by a preceding step.) From this contractive condition,

(44) ψ(d(Tun,Tz))≤ψ(G(un,z))−φ(G(un,z)),∀n≥n(b);

or, equivalently (combining with (42) above),

(45) $$\begin{array}{c} 0<\varphi \left(b\right)\leq \psi \left(b\right)-\psi \left(d\left(Tu_{n},Tz\right)\right),\;\forall n\geq n\left(b\right). \end{array}$$

Note that, as a consequence, *d*(*Tu*_*n*_, *Tz*) < *b*, for all *n* ≥ *n*(*b*). Passing to limit as *n* → *∞* and taking (37) into account, yields *φ*(*b*) ≤ *ψ*(*b*) − *ψ*(*b* − 0). This, however, contradicts the choice of (*ψ*, *φ*), so that *z* = *Tz*. The proof is complete.

In particular, when *ℛ* is transitive, this result is comparable with the one in Turinici [11]. Note that further extensions of these facts are possible, in the realm of triangular symmetric spaces, taken as in Hicks and Rhoades [16]; or, in the setting of partial metric spaces, introduced under the lines in Matthews [17]; we will discuss them elsewhere.

## 5. Further Aspects

Let in the following (*X*, *d*, *ℛ*) be a relational metric space; and let *T* be a self-map of *X*. Technically speaking, Theorem 7 that we just exposed consists of three substatements; according to the alternatives of our main result we already listed. For both practical and theoretical reasons, it would be useful to evidentiate them; further aspects involving the obtained facts are also discussed.

Before doing this, let us remark that the condition

(e01) *ℛ* is locally finitely transitive and nonidentical

appears as a particular case of (d01). On the other hand, (d01) is not deductible from (e01). In fact, (d01) has nothing to do with the points of

(e02) *X* ^*c*^(*T*, *ℛ*) : = *X*∖*X*(*T*, *ℛ*) = {*x* ∈ *X*; (*x*, *Tx*) ∉ *ℛ*}.

So, even if the restriction of *ℛ* to *X* ^*c*^(*T*, *ℛ*) is arbitrarily taken, (d01) may hold. On the other hand, (e01) cannot hold whenever *X* ^*c*^(*T*, *ℛ*) admits a denumerable subset *Y* such that the restriction of *ℛ* to *Y* is not finitely transitive; and this proves our assertion.

We may now pass to the particular cases of Theorem 7 with practical interest.

**Case 1**

As a direct consequence of Theorem 7, we get the following.

**Theorem 8**

Assume that *T* is *ℛ*-semiprogressive, *ℛ*-increasing, and Meir-Keeler (*d*, *ℛ*; *G*)-contractive, for some *G* ∈ *𝒢*. In addition, let *ℛ* be finitely semirecurrent nonidentical,  *X* be (a-o,  *d*)-complete, and one of the conditions below holds:

(i1) *T* is (a-o,  *d*)-continuous;
(i2) *ℛ* is (a-o,  *d*)-almost-self-closed and *G* ∈ *𝒢*_1_ : = {*A*_1_, *B*_2_, *B*_4_, *C*_1_}.

Then *T* is a globally strong Picard operator (modulo (*d*, *ℛ*)).

The following particular cases of this result are to be noted.

(1-1) Let *σ*(·) be a function in *ℱ*(*X* × *X*, *R*_+_); and *𝒮* denote the associated relation: [*x𝒮y* if and only if *σ*(*x*, *y*) ≥ 1]. Then, if we take *ℛ* : = *𝒮* and *G* = *A*_1_, the alternative (i1) of Theorem 8 includes the related statement in Berzig and Rus [18]. By the previous remark, this inclusion is—at least from a technical viewpoint—effective, but, from a logical perspective, it is possible that the converse inclusion be also true. Finally, the alternative (i2) of Theorem 8 seems to be new.
(1-2) Suppose that *ℛ* = *X* × *X* (i.e., *ℛ* is the* trivial relation* over *X*). Then, Theorem 8 is comparable with the main results in Włodarczyk and Plebaniak [19–22], based on contractive type conditions involving generalized pseudodistances. However, none of these is reducible to the remaining ones; we do not give details.

**Case 2**

As another consequence of Theorem 7, we have the following statement (with practical value).

**Theorem 9**

Assume that *T* is *ℛ*-semiprogressive, *ℛ*-increasing, and (*d*, *ℛ*; *G*, *φ*)-contractive, for some *G* ∈ *𝒢* and a certain Meir-Keeler admissible function *φ* ∈ *ℱ*(*re*)(*R*_+_). In addition, let *ℛ* be finitely semirecurrent nonidentical,  *X* be (a-o,  *d*)-complete, and one of the conditions below holds:

(j1) *T* is (a-o,  *d*)-continuous;
(j2) *ℛ* is (a-o,  *d*)-almost-self-closed.

Then *T* is a globally strong Picard operator (modulo (*d*, *ℛ*)).

The following particular cases of this result are to be noted.

(2-1) Suppose that *ℛ* = *X* × *X* (= the trivial relation over *X*) and *G* = *A*_1_. Then, Theorem 9 is comparable with the main results in Włodarczyk et al. [23, 24], based on contractive type conditions like
  (e03) diam⁡(*T*(*Y*)) ≤ *φ*(diam⁡(*Y*)), for all *Y* ∈ CB(*X*).

(Here, CB(*X*) is the class of all (nonempty) closed bounded subsets of *X*.) Clearly, this condition is stronger than the one we already used in Theorem 9. On the other hand, (e03) is written in terms of generalized pseudodistances. Hence, direct inclusions between these results are not in general available; we do not give details.

(2-2) Suppose that *ℛ* = *X* × *X*; and *φ* ∈ *ℱ*(*re*)(*R*_+_) is BWM-admissible (i.e., it is either Boyd-Wong admissible or Matkowski admissible). Then, if *G* = *A*_1_, Theorem 9 includes the Boyd-Wong result [13] when *φ* is Boyd-Wong admissible; and, respectively, the Matkowski's result [14] when *φ* is Matkowski admissible. Moreover, when *G* = *C*_2_, Theorem 9 includes the result in Leader [25].
(2-3) Suppose that *ℛ* is an order on *X*. Then, Theorem 9 includes the results in Agarwal et al. [26]; see also O'Regan and Petruşel [27].

**Case 3**

As a final consequence of Theorem 7, we have

**Theorem 10**

Assume that the self-map *T* is *ℛ*-semiprogressive, *ℛ*-increasing, and (*d*, *ℛ*; *G*, (*ψ*, *φ*))-contractive, for a certain *G* ∈ *𝒢* and some pair (*ψ*, *φ*) of generalized altering functions in *ℱ*(*R*_+_). In addition, let *ℛ* be finitely semirecurrent nonidentical,  *X* be (a-o,  *d*)-complete, and one of the conditions below holds:

(k1) *T* is (a-o,  *d*)-continuous;
(k2) *ℛ* is (a-o,  *d*)-almost-self-closed.

Then *T* is a globally strong Picard operator (modulo (*d*, *ℛ*)).

The following particular cases of this result are to be noted.

(3-1) Let *α*(·),  *β*(·) be a couple of functions in *ℱ*(*X* × *X*, *R*_+_); and *𝒜*,  *ℬ* stand for the associated relations:
  (46) $$\begin{array}{c} x𝒜y\;\text{iff}\;\alpha \left(x,y\right)\leq 1;\;\;xℬy\;\text{iff}\;\beta \left(x,y\right)\geq 1. \end{array}$$

Then, if we take *ℛ* : = *𝒜*∩*ℬ* and *G* ∈ *𝒢*, this result includes (cf. Lemma  1) the one in Berzig et al. [28], based on global contractive conditions like

(47) ψ(d(Tx,Ty))≤α(x,y)ψ(d(x,y))−β(x,y)φ(d(x,y)), ∀x,y∈X;

referred to as *T* is (*αψ*, *βφ*)-*contractive*. In particular, when *G* = *A*_1_, this last result reduces to the one in Berzig and Karapınar [7]; which, in turn, extends the one due to Samet et al. [29]; hence, so does Theorem 10 above.

(3-2) Let (*Y*, *d*) be a metric space; and *T* be a self-map of *Y*. Given *p* ≥ 2, let {*A*_1_,…, *A*_*p*_} be a finite system of closed subsets of *Y* with

(e04) *T*(*A*_*i*_)⊆*A*_*i*+1_, for all *i* ∈ {1,…, *p*} (where *A*_*p*+1_ = *A*_1_).

Define a relation *ℛ* over *Y* as

(e05) *ℛ* = (*A*_1_ × *A*_2_)∪⋯∪(*A*_*p*_ × *A*_*p*+1_);

then, put *X* = *A*_1_ ∪ ⋯∪*A*_*p*_. Clearly, *T* is a self-map of *X*; and the relation *ℛ* is *p*-semirecurrent at each orbital admissible point of *X*(*T*, *ℛ*). The corresponding version of Theorem 10 includes the related statement in Berzig et al. [28].

It is to be stressed that this last construction may be also attached to the setting of Case 2. Then, the corresponding version of Theorem 9 extends in a direct way some basic results in Kirk et al. [30].

Finally, we should remark that none of these particular theorems may be viewed as a genuine extension for the fixed point statement due to Samet and Turinici [6]; because, in the quoted paper, *ℛ* is not subjected to any kind of (local or global) transitive type requirements. Further aspects (involving the same general setting) may be found in Berzig [31].
